# “Freeze, Don’t Move”: How to Arrest a Suspect in Heart Failure – A Review on Available GRK2 Inhibitors

**DOI:** 10.3389/fcvm.2016.00048

**Published:** 2016-12-06

**Authors:** Daniela Sorriento, Michele Ciccarelli, Ersilia Cipolletta, Bruno Trimarco, Guido Iaccarino

**Affiliations:** ^1^Department of Advanced Biomedical Sciences, University of Naples Federico II, Naples, Italy; ^2^Department of Medicine, Surgery and Dentistry “Scuola Medica Salernitana”, University of Salerno, Baronissi, SA, Italy

**Keywords:** GRK2, heart failure, catalytic activity, gene therapy, peptide-based drug, GRK2 interactome

## Abstract

Cardiovascular disease and heart failure (HF) still collect the largest toll of death in western societies and all over the world. A growing number of molecular mechanisms represent possible targets for new therapeutic strategies, which can counteract the metabolic and structural changes observed in the failing heart. G protein-coupled receptor kinase 2 (GRK2) is one of such targets for which experimental and clinical evidence are established. Indeed, several strategies have been carried out in place to interface with the known GRK2 mechanisms of action in the failing heart. This review deals with results from basic and preclinical studies. It shows different strategies to inhibit GRK2 in HF *in vivo* (βARK-ct gene therapy, treatment with gallein, and treatment with paroxetine) and *in vitro* (RNA aptamer, RKIP, and peptide-based inhibitors). These strategies are based either on the inhibition of the catalytic activity of the kinase (“Freeze!”) or the prevention of its shuttling within the cell (“Don’t Move!”). Here, we review the peculiarity of each strategy with regard to the ability to interact with the multiple tasks of GRK2 and the perspective development of eventual clinical use.

## Introduction

Heart failure (HF) is the final phenotype of several degenerative conditions, which lead to the incapacity of the heart to pump enough blood to meet body’s demand, if they are not counteracted (1, 2): myocardial infarction, high blood pressure, arrhythmia, cardiomyopathy, congenital heart defects, heart valve disease, diabetes, alcohol abuse or illegal drug use, HIV/AIDS, thyroid disorders, radiation, and chemotherapy.

In the early stages of HF, cardiovascular homeostasis is maintained by several compensatory neurohormonal mechanisms and patients can remain asymptomatic for a long time. Then, the heart undergoes several changes, such as an increase in cardiac mass and alterations in the extracellular matrix, even if the cardiac function is still maintained (remodeling). The late stage of HF, which is due to a constant and long-term strain, is characterized by cardiac enlargement and a progressive decrease of the contractile function (3).

Several changes lead to the progressive loss of the contractile function and to the decreased responsiveness to the normal adrenergic control mechanisms (4): loss of myofilaments in cardiac myocytes (5), alterations in cytoskeletal proteins (5), alterations in excitation–contraction coupling (6), and desensitization of β-adrenergic signaling (7). Moreover, the failing cardiomyocyte is characterized by mitochondrial dysfunction with an altered ability to use metabolic substrates for the production of energetic compounds (8, 9). Recent studies have underlined the key role of mitochondria in the progression of the myocardial dysfunction and the metabolic remodeling, in the deficit of the cardiac energetics and the increased oxidative stress (10). This latter is due to an excessive production of reactive oxygen species (ROS) and plays a key role in the pathophysiology of the cardiac remodeling and the HF. Indeed, oxidative stress causes cellular dysfunction and damage, leading to the activation of pro-death signaling (11–14).

In the last decade, molecular biology and genetics have elucidated the key pathways that are involved in the development and the progression of HF and have identified specific molecules that could be potential targets for pharmacological approaches (15). In this context, G protein-coupled receptor kinase type 2 (GRK2) seems to be one of the main candidates.

## GRK2 in Heart Failure

**Figure 1:**
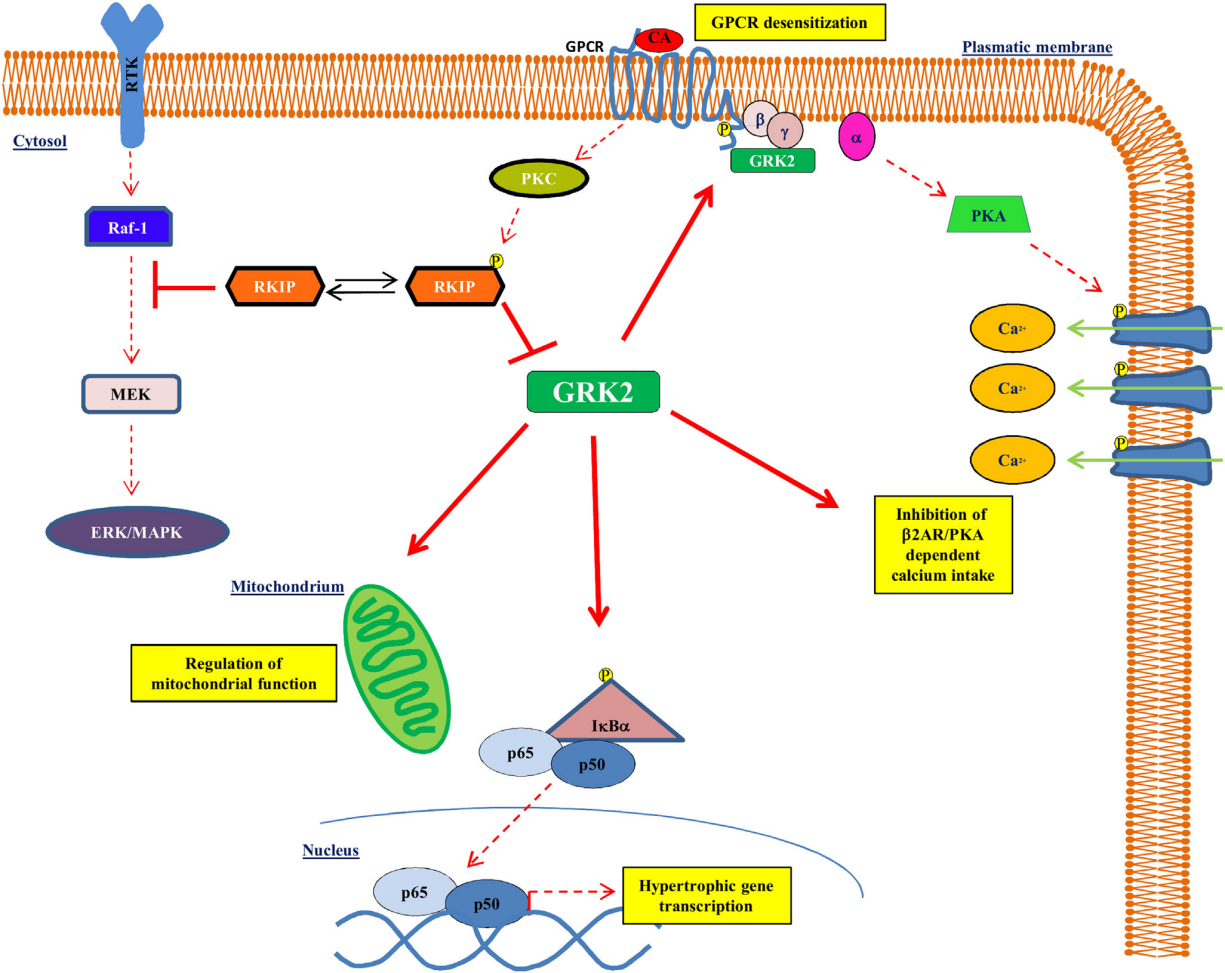
**GRK2 effects on intracellular signaling**. GRK2 exerts different effects within the cell affecting several intracellular signaling. Indeed, GRK2 regulates GPCR activation by receptor phosphorylation, thus affecting GPCR dependent phenotypes, such as regulation of calcium intake. GRK2 localizes to mitochondria and regulates mitochondrial function. Finally, GRK2 phosphorylates IκBα thus allowing NFκB nuclear translocation and transcription activity. Upon GPCR activation, RKIP is phosphorylated at Ser153 by PKC and inhibits GRK2.

G protein-coupled receptor kinase type 2 is a cytosolic enzyme that localizes to the plasma membrane, through the binding to the βγ subunits of activated G proteins (Gβγ), and regulates the activation of beta-adrenergic receptor (βAR) signaling (16, 17). Changes of kinase activity and expression play an important role in the development and maintenance of the cardiac hypertrophy and of HF (16, 18). In particular, GRK2 levels increase during left ventricular hypertrophy (19–24) and associate with a reduction of βAR signaling and with an impaired cardiac contractility (25). In the heart, besides βAR downregulation and desensitization, GRK2 interacts with different intracellular partners to regulate several cardiomyocyte functions. Indeed, the increase of GRK2 levels during chronic HF induces several changes: increase of cardiac insulin resistance, reduction of cardiac metabolic plasticity (18, 26), regulation of intracellular calcium homeostasis (27), and activation of NFκB signaling (24) (Figure 1). It has also been demonstrated in cell types different from cardiac myocytes that GRK2 activates the mitochondrial function (28, 29). Because of its molecular and functional complexity, it is not surprising that this molecule regulates both the function and the development of the cardiovascular system. Indeed, the genetic deletion of GRK2 is lethal since it leads to a wrong development of the cardiovascular system during prenatal life (30, 31). Moreover, the selective cardiac deletion of the kinase causes a prevalent eccentric remodeling in response to the chronic exposure to β adrenergic agonists (32).

## Freeze/Do Not Move

It is now validated the proof of concept that GRK2 regulates several intracellular signaling pathways not only through the phosphorylation of specific substrates but also through protein–protein interactions independently from its catalytic activity (24, 29, 33–35). In this context, it is clear that potential approaches to inhibit the kinase effects could be countless. Indeed, it is possible to freeze GRK2 through the selective inhibition of its catalytic activity to modulate phosphorylation-dependent effects. It is also possible to disrupt GRK2 interactions with its substrates through the use of selective peptides. Given the recent findings on GRK2 subcellular localization, it is likely that the regulation of the kinase moving within the cell could be useful to control its effects, such as favoring its mitochondrial localization rather than plasma membrane translocation. Here, we discuss these issues and deal with known and potential approaches to freezing GRK2 in HF.

## Targeting GRK2 in Heart Failure

**Figure 2:**
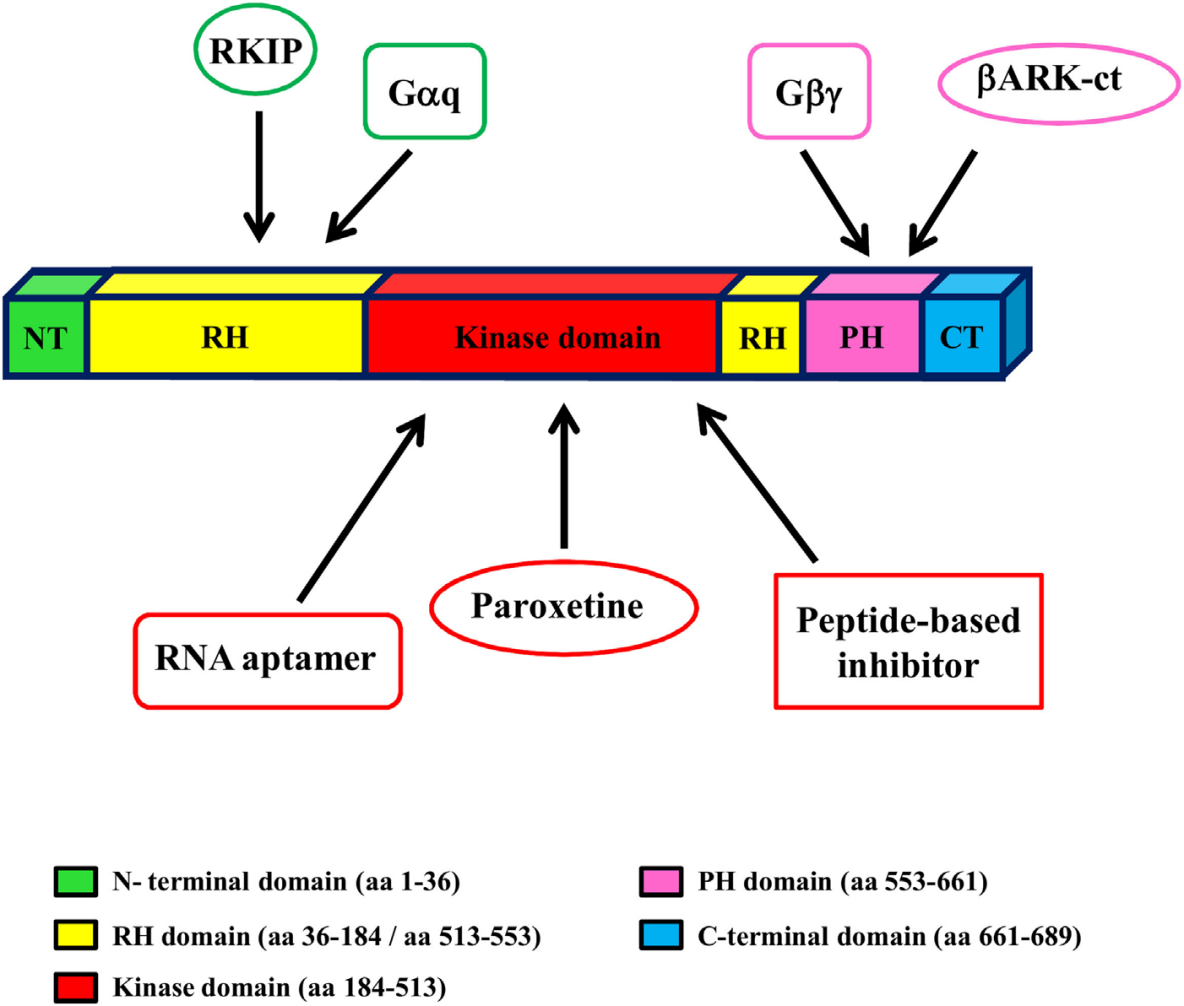
**GRK2 structure and inhibitors**. GRK2 has a central catalytic domain flanked by an N-terminal domain, including the RGS domain, and a carboxyl-terminal domain, including the catalytic domain and the PH domain. The binding site of GRK2 inhibitors is indicated by arrows.

Given the key role of GRK2 in the development and progression of cardiovascular diseases (CVD), including HF, targeting GRK2 could be an effective therapeutic strategy for HF. To date, several approaches have been evaluated to reach this aim in an animal model of HF (gene therapy, treatment with paroxetine and gallein, cardiac expression of a specific sequence of GRK2) (Figure 2).

### Gene Therapy

Gene therapy is a novel approach to treat and prevent diseases by changing the expression of target genes. Recently, this technique has been moved from the laboratory research to translational clinical trials for many diseases, such as severe combined immune deficiency, hemophilia, cancer, chronic granulomatous disorder, and neurodegenerative diseases (36).

For CVD, gene therapy has recently been proposed (37–39) mainly for the treatment of coronary artery disease, HF, and arrhythmias (40). In particular, gene therapy targets for HF are sarcoendoplasmic reticulum calcium-ATPase 2a (SERCA2a) and stromal-derived factor-1 (SDF-1), which are the actual objects of ongoing clinical trials (41, 42).

In addition to these targets, it has been demonstrated that several other genes are effective in the treatment of HF in animal models of disease. In this context, gene therapy has been used in preclinical studies to target GRK2 on the plasma membrane and to avoid βAR desensitization. This is achieved through the expression of βARKct that is mediated by the adenovirus (AD) or adeno-associated virus (AAV). βARKct resembles the carboxy-terminal domain of GRK2 that is responsible for its translocation to the plasma membrane and its binding to Gβγ. This strategy is effective in several models of CVD. Raake and colleagues used adeno-associated virus serotype 6 (AAV6) to express βARKct in a porcine model of HF (43). The Authors found that the long-term βARKct expression induced a significant amelioration of left ventricular hemodynamics and contractile function in pigs with HF compared to controls, which showed an impaired cardiac function.

The ventricular delivery of Adeno-βARKct in failing hearts of rabbits, using coronary catheterization, reversed ventricular dysfunction (44). These findings support the idea that gene therapy with βARKct could become an effective therapeutic strategy for HF. Viral vectors are commonly used for cardiovascular applications, including AD and AAV, which can infect non-dividing cells and transduce heart with good efficiency. Differences between the two DNA viruses regard the limited amount of DNA that AAVs can carry, and the high inflammatory response of the ADs, which limits the time of expression of the transgene. Therefore, limitations that prevent the use of AAV expressing βARKct in humans have still to be overcome. First of all, it is not completely known the full range of effects that viruses expressing βARKct can exert on GRK2 and also on other intracellular signalings. Indeed, it is known that βARKct is able to displace GRK2 from plasma membrane allowing its translocation to other compartments (29). In lipopolysaccharide (LPS)-treated macrophages, the adenoviral-mediated gene transfer of βARKct maintains macrophage functionality by inducing an earlier localization of GRK2 to mitochondria (29). Indeed, βARKct also blocks βγ signaling (45) and prevents cellular responses to important extracellular stimulants. These findings clearly demonstrate that βARKct, besides GRK2 inhibition in the plasma membrane, exerts multiple effects within the cell. Indeed, through the interaction with Gβγ, βARKct also inhibits Gβγ signaling that is involved in the regulation of cell proliferation and survival. Moreover, βARKct, by displacing GRK2 from the plasma membrane, allows the kinase to accumulate in other cellular compartments, where GRK2 can interfere with many other cellular functions. Therefore, it is likely that βARKct-based gene therapy in humans could provoke several side effects.

### Selective Inhibitory Drugs

A recent study shows that paroxetine, the selective serotonin reuptake inhibitor (SSRI), can inhibit GRK2 activity (46, 47). Thal and colleagues show that paroxetine binds the active site of GRK2 stabilizing the kinase domain in a novel conformation (46). Both *in vitro* in isolated cardiomyocytes and *in vivo* in mice, pretreatment with paroxetine potentiates isoproterenol effects on βAR-mediated contractility (46). Moreover, in wild-type mice with myocardial infarction, paroxetine significantly improves cardiac function (47). Paroxetine seems to be an efficient inhibitor of GRK2 with selectivity over other GRKs even if it is still unknown its selectivity over other kinases and its side effects *in vivo* in other tissues. A major limitation for the use of this drug is the very high dosage at which it is effective to inhibit the kinase. Indeed, the effective doses exceed those approved for the use of paroxetine in humans, making unavoidable effects on the central nervous system. It is most likely that paroxetine will never be used in humans for the treatment of cardiac dysfunction in HF.

### Non-Selective Inhibitory Drugs

Gallein is a novel small molecule that selectively blocks Gβγ-binding interactions, including the one with GRK2. It has been shown that gallein reduces the recruitment of GRK2 on the plasma membrane and enhances contractility in isolated adult mouse cardiomyocytes in response to a βAR agonist (48). In a mouse model of HF due to isoproterenol injections, the treatment with gallein prevents HF and reduces GRK2 expression (48). These data suggest that gallein could be a promising therapeutic drug for the treatment of HF. However, gallein is a specific inhibitor of Gβγ rather than GRK2. Thus, it is likely that this molecule affects other intracellular signalings like βARKct.

### Cardiac Overexpression of a Specific Domain of GRK2

Since it has been shown that the Regulator of G Protein Signaling (RGS) domain of GRK2 interacts with Gαq and inhibits it *in vitro*, transgenic mice with cardiac-specific expression of the RGS domain of GRK2 have been generated and subjected to cardiac damage in response to pressure overload. These mice show less hypertrophy and less adverse structural remodeling compared with controls (49). In this case, it appears that the beneficial effect is more on Gαq inhibition rather than on GRK2 inhibition. These data confirm previous works of the group of Gerard Dorn, who was the first to exploit Gαq as a mechanism of cardiac hypertrophy (50). Thus, RGS domain of GRK2 could be used as a prototype for the development of effective drugs to prevent cardiac hypertrophy.

## Potential Strategies to Inhibit GRK2 in Heart Failure

Other potential inhibitors have been identified and tested *in vitro* in cultured cells [RNA aptamers, Raf kinase inhibitor protein (RKIP), and peptide inhibitors] (Figure 2), but their effectiveness has never been tested *in vivo* in animal models of HF. Thus, they could become therapeutic drugs for HF *in vivo* even if further experiments are necessary to verify this hypothesis.

### RNA-Based Inhibitors

RNA aptamers have been developed to inhibit GRK2 through systematic evolution of ligands by exponential enrichment (SELEX). Among them, C13 binds GRK2 with a high affinity and inhibits GRK2-dependent rhodopsin phosphorylation *in vitro* (51). C13 can stabilize GRK2 in an inactive conformation through multiple interactions in the active site pocket of the kinase domain (52). In particular, the positioning of an adenine nucleotide in the ATP-binding pocket and the interactions with the basic αF–αG helicoidal regions of the GRK2 kinase domain are mainly involved in the kinase inhibition. The use of aptamers is limited to *in vitro* studies but could be converted into small inhibitors through an aptamer-displacement assay (53). Thus, this approach could be potentially transferred to the clinical scenario, even if further studies are necessary to reach this aim.

### Physiological Inhibitors: RKIP

Raf kinase inhibitor protein modulates several key intracellular signaling, including the signaling cascades of ERK, NFκB, glycogen synthase kinase-3β (54–56). It has been shown that RKIP is also a physiological inhibitor of GRK2 (57). After the activation of G protein-coupled receptors, RKIP dissociates from Raf-1 to associate with GRK2. This switch is due to RKIP dimerization (58) that is regulated by PKC-mediated phosphorylation at Ser-153 (57). RKIP binds GRK2 in the amino-terminal domain. In cardiomyocytes, the downregulation of RKIP inhibits beta-adrenergic signaling and contractile activity (57). This evidence suggests that this physiological mechanism of inhibition of GRK2 could be useful for the treatment of CVD. However, the enthusiasm of this discovery is cooled by the poor selectivity of this small protein on kinase activity since RKIP also affects several intracellular signaling pathways.

### Peptide-Based Inhibitors

The design and the synthesis of peptide-based compounds have spread in the last decade (59). The use of peptides as therapeutic drugs has some limitations, including the parenteral route of administration since peptides are not well absorbed in the gastrointestinal tract. Moreover, peptides do not usually cross plasma membrane and are rapidly metabolized by proteolytic enzymes. However, compared to synthetic small molecules, peptides are less toxic, more selective, and they do not accumulate in organs. Their rapid degradation makes them less harmful, and their degradation products are simply amino acids and should not have toxic effects (60). Considering these advantages, it is not surprising that there are many peptide-based drugs available on the market (59), such as receptor agonists and antagonists, peptide hormones and analogs, and HIV protease inhibitors (61). Several peptide inhibitors of GRK2 have been developed, modeled on the structure of the kinase. It has been demonstrated that the inhibition of GRK2 by GRKInh, a peptide inhibitor of the kinase (62), could counteract the dysfunctional metabolism of HF in a transgenic model of myocardium-specific expression of fatty acid synthase (FASN) (63). Anis et al. (64) demonstrated that myristyl or lauryl glycine derivatives of short peptides derived from the HJ loop of GRK2, KRX-683_107_, and KRX-683_124_ are potent inhibitors of the kinase and exert hypoglycemic effects in animal models of type 2 diabetes. We have recently found that non-acylated derivatives of KRX-683_107_ and KRX-683_124_ (peptides **2** and **3**) selectively inhibit GRK2 *in vitro* (65). *In vivo* in hypertensive rats, the infusion of peptide **3** for 30 days ameliorates GRK2-dependent insulin resistance and IRS1 tyrosine phosphorylation (66). Moreover, the intracardiac injection of this peptide reduces phenylephrine- or hypertension-induced left ventricular hypertrophy (24). Thus, it is likely that this peptide could be effective to ameliorate the cardiac morphology and function in the failing heart.

## Suggestions for Future Directions

### The New Generation of Peptide Inhibitors: Cyclic Peptides

To reach better results, cyclic peptides have recently been designed. These are polypeptide chains taking cyclic ring structure by linking the two ends of the peptide with an amide bond or other chemically stable bonds. Cyclic peptides have a better biological activity compared to their linear counterparts due to their conformational rigidity (67), which allows a selective binding with their targets. Moreover, their cyclic structure makes peptides resistant to hydrolysis by exopeptidases (due to the lack of both amino and carboxyl termini) and endopeptidases (since the structure is less flexible than linear peptides). Some cyclic peptides can autonomously cross plasma membrane, thus avoiding the need of a vehicle for internalization, such as HIV-1 Tat protein and Penetratin, which increases peptide size.

In particular, cyclic compounds have been designed, which are modeled on the conformation of the HJ loop within the X-ray structure of GRK and are based on the structure of the above-described KRX-683_124_. One of these compounds, the cyclic peptide **7**, can inhibit GRK2 activity and is more active than its linear precursor. In cultured cells, this peptide confirms its potentiality and specificity as a GRK2 inhibitor (68). Thus, this cyclic peptide has a great potentiality to be translated to clinical trials.

### Balance between GRK2 Degradation and Synthesis

**Figure 3:**
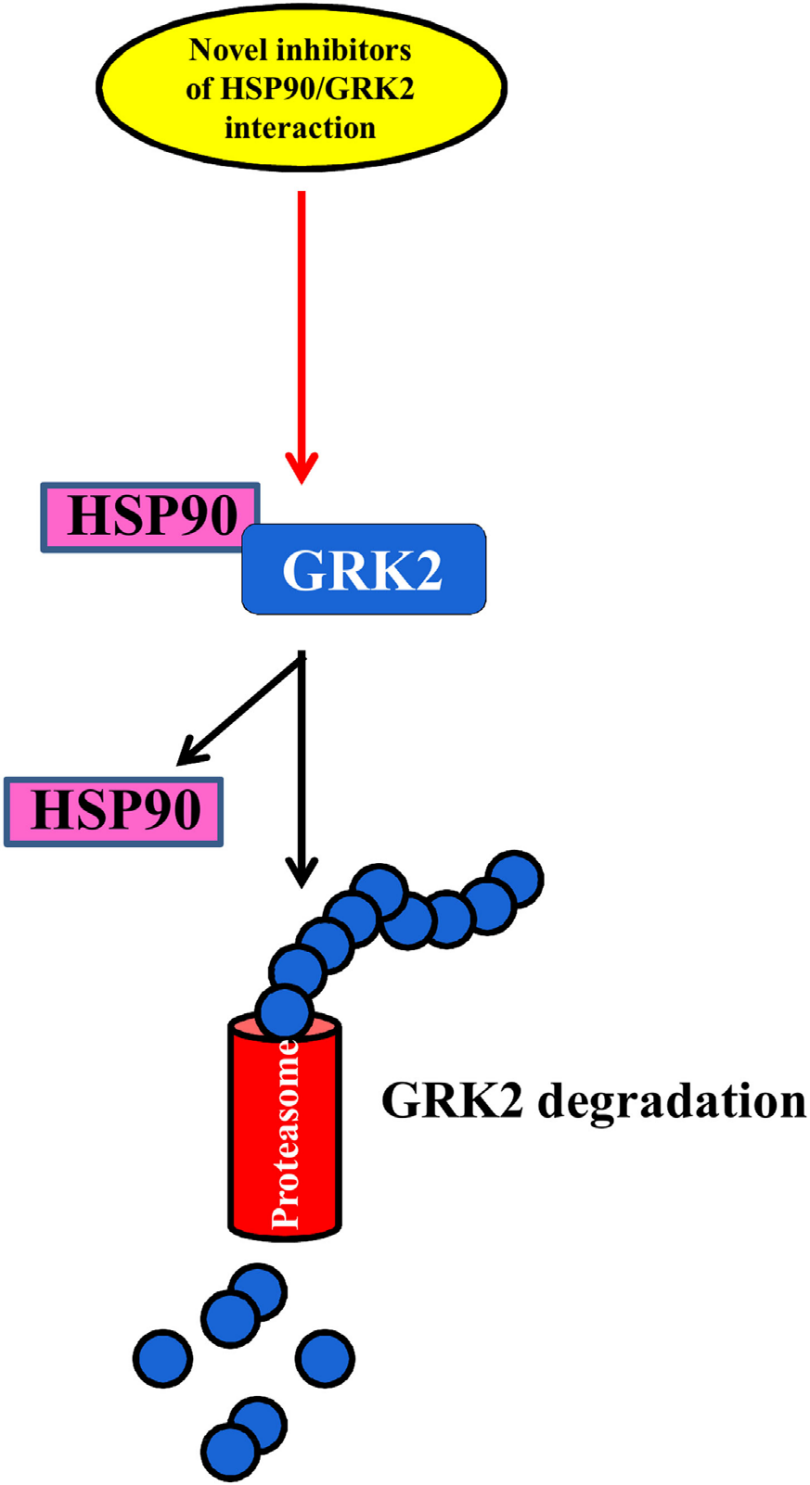
**The balance between GRK2 synthesis and degradation**. The identification of novel inhibitors of HSP90–GRK2 interaction could be useful to induce GRK2 degradation by proteasome vs. its synthesis thus reducing the deleterious effects of the kinase in cardiovascular diseases.

The regulation of the balance between GRK2 degradation and its synthesis could be an effective approach to reducing GRK2 levels in several diseases (69, 70) (Figure 3). The degradation of GRK2, for instance, that occurs *via* the proteasome (69) can be prevented by its interaction with Hsp90, which stabilizes the correct folding of the protein (71). Thus, NMR analysis of this complex could help to develop new inhibitors that can reduce the interaction between HSP90 and GRK2, leading to kinase degradation in pathological conditions.

### Regulation of the Subcellular Localization of GRK2

**Figure 4:**
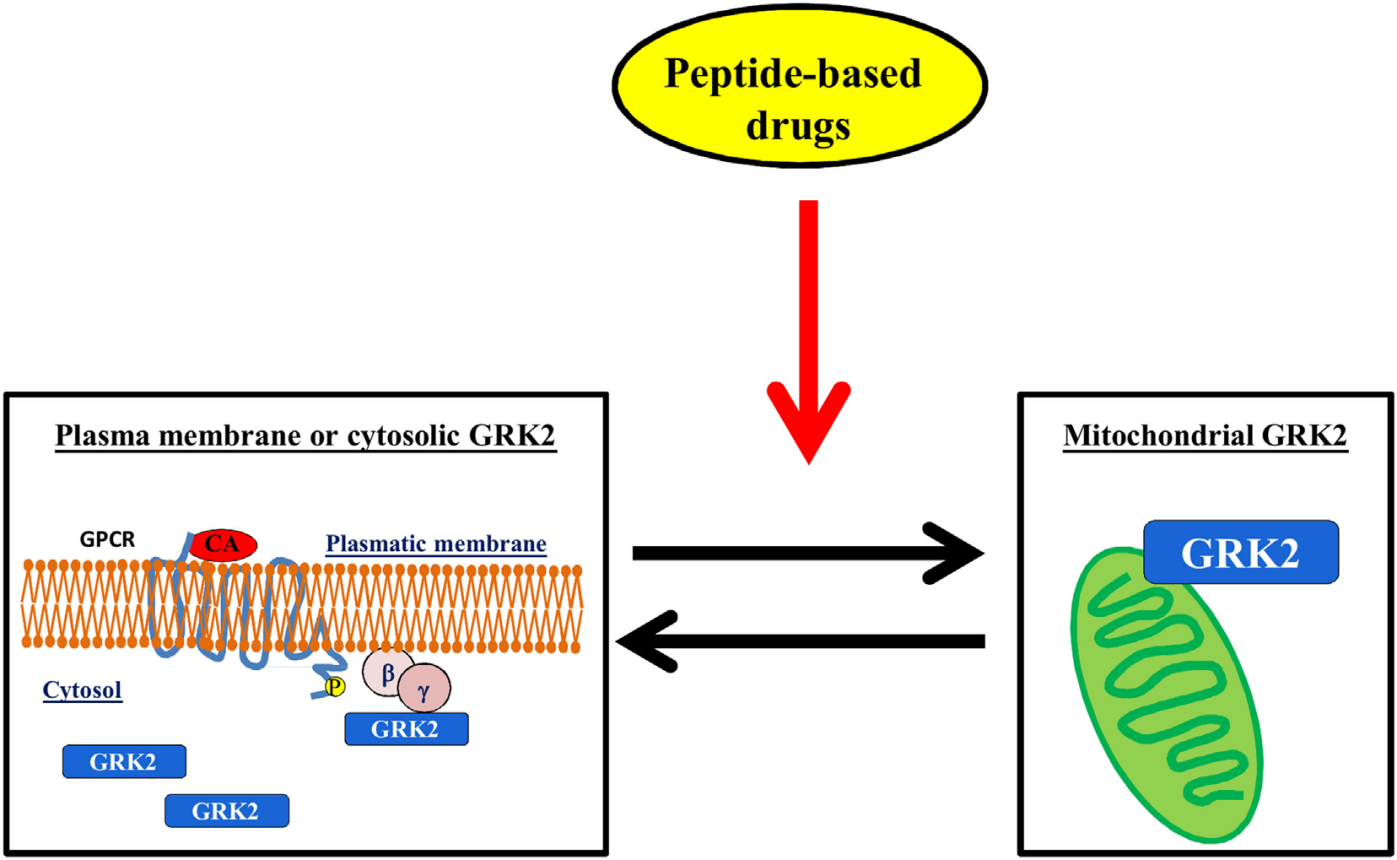
**The regulation of GRK2 localization within the cell**. The possibility to induce mitochondrial localization of GRK2 could increase cell metabolism thus favoring the advantageous effects of the kinase vs. the deleterious effects on plasma membrane or cytosol.

The regulation of GRK2 localization within the cell could be a useful target for diseases. Indeed, it is emerging that GRK2 exerts different effects within the cell, which depend on its localization, cell type, stimuli, and physiopathological context (28, 33, 34, 72). In particular, several stressors increase the levels of GRK2 in mitochondria, in an ERK- and HSP90-dependent mechanism (73). The effects of such accumulation are still the object of investigation since opposite results in the literature show either a protective mechanism (28, 29, 74) or the acceleration of unfavorable processes (73). Nevertheless, given the established notion that the accumulation of GRK2 in plasma membrane inhibits GPCR signaling or its binding with cytosolic substrates activates pro-death signaling, the possibility to modulate GRK2 accumulation within specific organelles might in the future pose the strategy to regulate kinase effects in pathological conditions (Figure 4).

## Conclusion

To date, several approaches have been developed to inhibit GRK2 activity, which are based on different molecular mechanisms. Most of them are far from clinical applications, but they will be helpful for the development of novel inhibitors (βARK-ct, paroxetine, gallein, RNA aptamers). To date, the most feasible approach, which could easily be translated to clinical trials, seems to be the peptide-based drug, and cyclic peptides are the most promising. However, advancements in the knowledge of the multiple roles of GRK2 within the cell in HF will allow the identification of more selective inhibitors.

## Author Contributions

DS and GI conceived and designed the work. MC, EC, BT, DS, and GI drafted the work and revisited it critically.

## Conflict of Interest Statement

The authors declare that the research was conducted in the absence of any commercial or financial relationships that could be construed as a potential conflict of interest.
